# Supplementary material for: Prediction of Prognosis and Chemotherapeutic Sensitivity Based on Cuproptosis-Associated lncRNAs in Cervical Squamous Cell Carcinoma and Endocervical Adenocarcinoma
Source: Genes (Basel). 2023 Jun 30;14(7):1381. doi: 10.3390/genes14071381 (PMC10379127; doi:10.3390/genes14071381)
Supplement: Supplementary file 1 [file genes-14-01381-s001.zip › genes-2466642-supplementary.pdf]

Prediction of Prognosis and Chemotherapeutic Sensitivity Based on Cuproptosis-Associated lncRNAs in Cervical Squamous Cell Carcinoma and Endocervical Adenocarcinoma

Jianghong Zhou [1,2,†], Lili Xu [1,†], Hong Zhou [1], Jingjin Wang [1] and Xiaoliang Xing [2,*]

1 Department of Gynecology, Department of Obsterics and Gynecology, Zhuzhou Hospital Affiliated to Xiangya school of Medicine, Central South University, Zhuzhou 412007, China; zhoujianghong84@163.com (J.Z.); xll1230404@126.com (L.X.); 15576518668@163.com (H.Z.); wjjzzzxyy@163.com (J.W.)
2 School of Public Health and Laboratory Medicine, Hunan University of Medicine, Huaihua 418000, China
* Correspondence: xiaoliangxinghnm@126.com
† These authors contributed equally to this work.

Supplementary information: 1 table and 3 figures.

Table S1. OS related CRLs filtered by Univariate and multivariate Cox regression analysis.

Cox	Term	P	HR	HRlower	HRupper
	AC002064.1	0.018	0.55	0.34	0.90
	AC004080.1	0.033	0.58	0.36	0.96
	AC008771.1	0.007	0.52	0.32	0.84
	AC010864.1	0.013	0.55	0.34	0.88
	AC011468.3	0.000	0.40	0.24	0.65
	AC012306.2	0.011	1.85	1.15	2.97
Uni-	AC024337.2	0.004	0.50	0.31	0.81
	AC063943.1	0.028	0.58	0.36	0.94
	AC104123.1	0.037	1.66	1.03	2.68
	AC107464.2	0.003	0.47	0.29	0.78
	AC139530.1	0.000	0.38	0.23	0.63
	AF165147.1	0.000	0.40	0.25	0.66
	AL031673.1	0.001	0.42	0.26	0.69

AL049543.1	0.035	1.65	1.04	2.63
AL136295.6	0.000	0.39	0.24	0.64
AL137026.1	0.029	0.58	0.36	0.95
AL162574.1	0.031	0.48	0.25	0.94
AL353593.1	0.015	1.82	1.13	2.95
AL592494.1	0.019	0.43	0.21	0.87
AP001020.2	0.044	1.62	1.01	2.59
AP001350.1	0.004	2.06	1.26	3.37
B3GALT1-AS1	0.029	0.55	0.32	0.94
BAIAP2-DT	0.009	1.89	1.17	3.04
CNNM3-DT	0.001	0.42	0.26	0.69
FAM157C	0.013	1.83	1.14	2.94
GNAS-AS1	0.001	2.24	1.39	3.63
KCNMB2-AS1	0.038	1.65	1.03	2.64
LACTB2-AS1	0.020	0.57	0.35	0.92
LINC00460	0.002	2.21	1.35	3.61
LINC01206	0.012	0.54	0.34	0.87
LINC02166	0.007	0.51	0.31	0.83
LINC02253	0.010	0.53	0.33	0.86
LINC02325	0.004	0.49	0.31	0.80
LINC02332	0.017	0.56	0.35	0.90
LINC02356	0.001	0.42	0.26	0.69
LINC02551	0.004	2.01	1.25	3.25
LINC02610	0.031	0.59	0.37	0.95
LNCOG	0.017	1.77	1.11	2.83
MAILR	0.025	1.71	1.07	2.74
MAP4K3-DT	0.025	1.73	1.07	2.79
PYCARD-AS1	0.013	0.54	0.33	0.88
SNHG30	0.003	0.48	0.30	0.78

	SNHG9	0.004	0.48	0.29	0.80
	SOX21-AS1	0.003	0.48	0.30	0.79
	AC002064.1	0.629	0.74	0.21	2.56
	AC004080.1	0.117	0.33	0.08	1.33
	AC008771.1	0.175	0.51	0.19	1.35
	AC010864.1	0.995	1.00	0.30	3.30
	AC011468.3	0.714	0.82	0.29	2.35
	AC012306.2	0.138	2.32	0.76	7.06
	AC024337.2	0.870	1.11	0.31	3.95
	AC063943.1	0.102	2.30	0.85	6.26
	AC104123.1	0.172	1.94	0.75	5.05
	AC107464.2	0.249	2.34	0.55	9.90
	AC139530.1	0.813	0.86	0.24	3.09
	AF165147.1	0.092	0.32	0.08	1.21
	AL031673.1	0.951	1.03	0.36	2.93
Mul-	AL049543.1	0.902	1.07	0.39	2.93
	AL136295.6	0.485	1.51	0.47	4.83
	AL137026.1	0.374	1.71	0.52	5.56
	AL162574.1	0.352	0.57	0.17	1.87
	AL353593.1	0.973	0.98	0.28	3.45
	AL592494.1	0.564	0.61	0.11	3.25
	AP001020.2	0.099	2.34	0.85	6.44
	AP001350.1	0.841	0.90	0.32	2.52
	B3GALT1-AS1	0.216	0.45	0.12	1.60
	BAIAP2-DT	0.996	1.00	0.26	3.81
	CNNM3-DT	0.592	1.38	0.42	4.53
	FAM157C	0.072	2.69	0.92	7.92
	GNAS-AS1	0.347	0.57	0.18	1.84
	KCNMB2-AS1	0.854	1.09	0.42	2.85

LACTB2-AS1	0.138	0.44	0.15	1.30
LINC00460	0.157	2.48	0.70	8.75
LINC01206	0.842	0.89	0.29	2.78
LINC02166	0.591	0.75	0.26	2.17
LINC02253	0.221	0.51	0.17	1.51
LINC02325	0.538	0.71	0.24	2.11
LINC02332	0.461	0.66	0.22	1.98
LINC02356	0.832	0.89	0.29	2.72
LINC02551	0.402	1.55	0.56	4.29
LINC02610	0.484	0.66	0.20	2.14
LNCOG	0.396	0.61	0.20	1.90
MAILR	0.370	1.60	0.57	4.49
MAP4K3-DT	0.950	1.04	0.31	3.43
PYCARD-AS1	0.549	0.77	0.32	1.83
SNHG30	0.837	1.12	0.37	3.40
SNHG9	0.180	2.13	0.71	6.44
SOX21-AS1	0.058	0.30	0.09	1.04

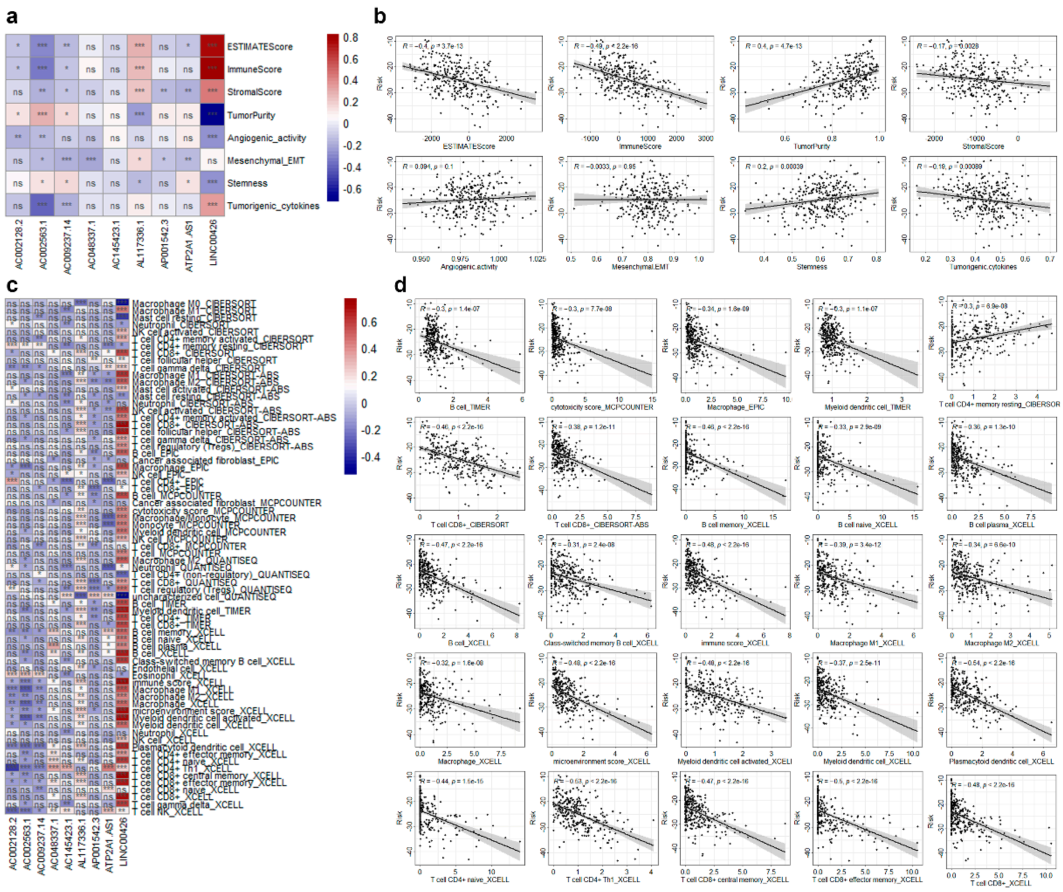

Figure S1. Correlation of nine CRLs and risk score with the immune characteristic.

a, Correlation analysis for the tumor microenvironment and tumor-related scores with nine CRLs. **b,** Correlation analysis for the tumor microenvironment and tumor-related scores with risk score. **c,** Correlation analysis for the immune score with nine CRLs. **d,** Correlation analysis for the immune score and with risk score.

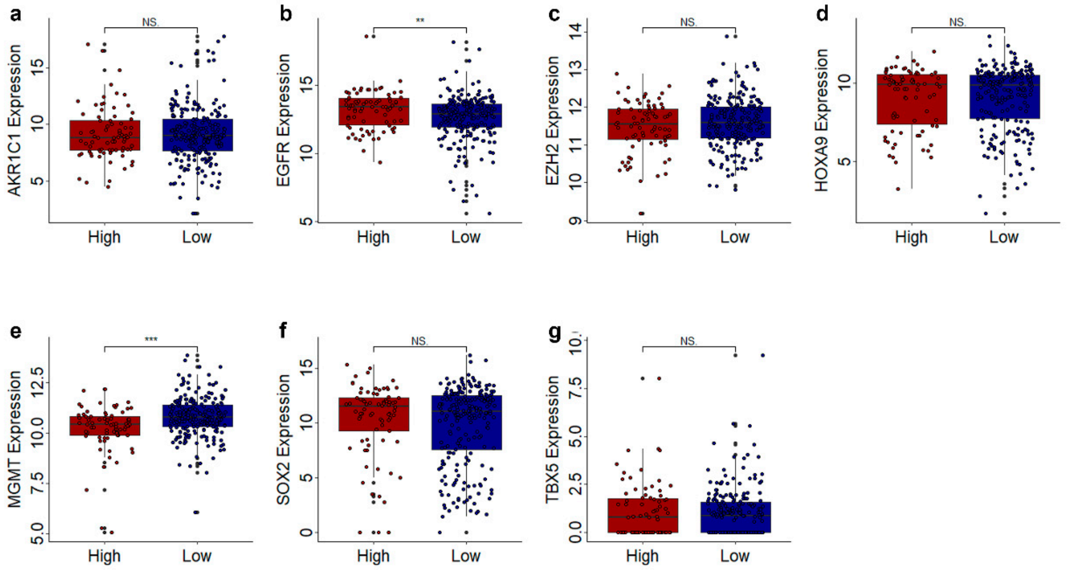

Figure S2. Expression of seven CRSGs between high- and low-risk score.

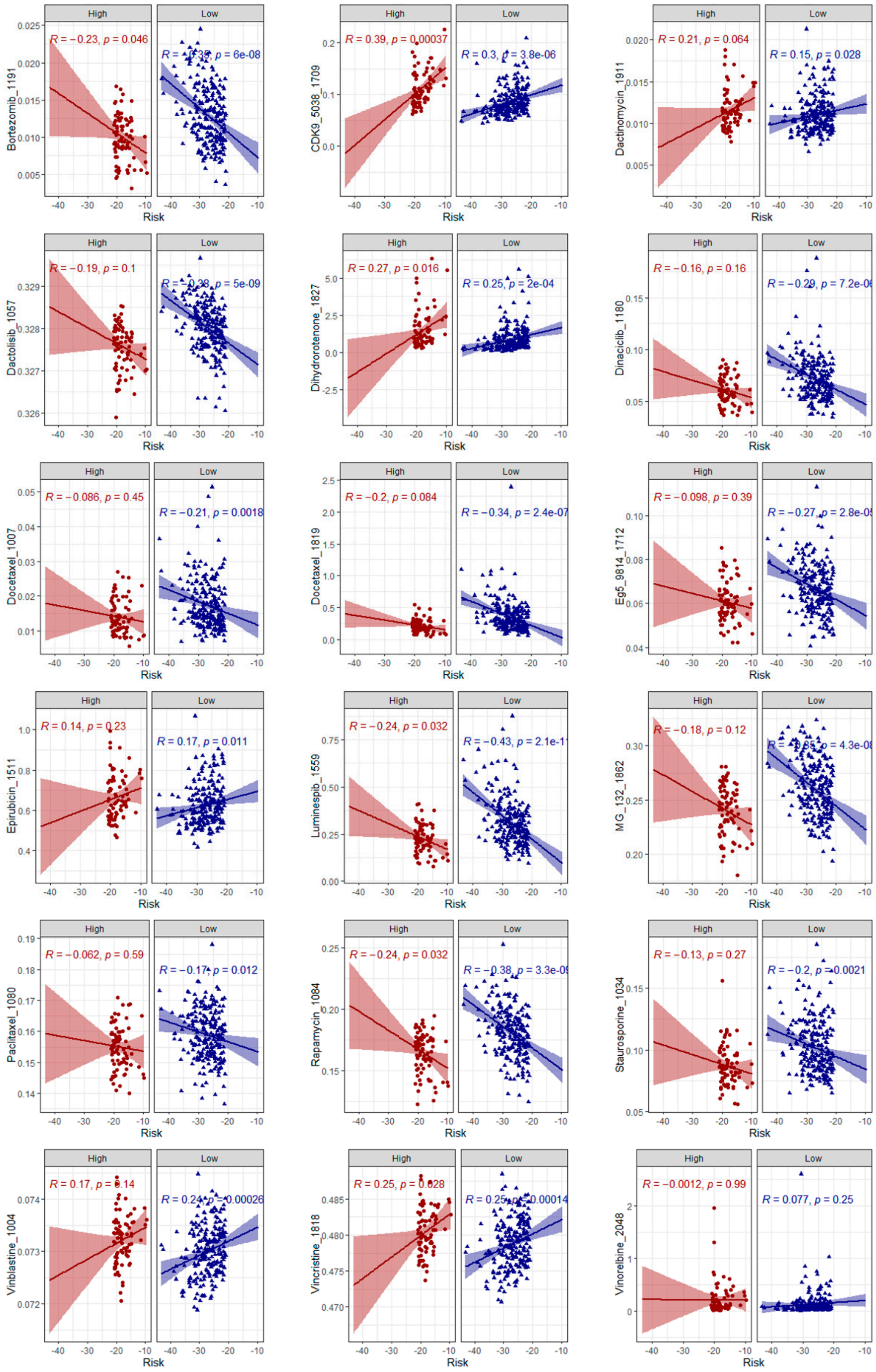

Figure S3. Correlation of risk score and drug sensitivity in different group.
